# Access to ground truth at unconstrained size makes simulated data as indispensable as experimental data for bioinformatics methods development and benchmarking

**DOI:** 10.1093/bioinformatics/btac612

**Published:** 2022-09-08

**Authors:** Geir Kjetil Sandve, Victor Greiff

**Affiliations:** Department of Informatics, University of Oslo, 0316 Oslo, Norway; Centre of Bioinformatics, University of Oslo, 0316 Oslo, Norway; UiORealArt convergence environment, University of Oslo, 0316 Oslo, Norway; Department of Immunology, University of Oslo and Oslo University Hospital, 0316 Oslo, Norway

## 1 Introduction

The author instructions of this journal (*OUP Bioinformatics*) include several detailed requirements for manuscripts to be considered for publication (https://academic.oup.com/bioinformatics/pages/instructions_for_authors). Such requirements clarify expectations for authors and establish important peer-review standards. Importantly, it also allows for an open discussion of these requirements, which for a leading journal such as *Bioinformatics* contributes to shaping the fields of computational biology and bioinformatics. One of the requirements is that manuscripts presenting new methodology must include ‘actual biological data’ as opposed to simulated data. We find this requirement potentially counterproductive for several reasons and argue for emphasizing the complementarity of simulated and experimental data.

Before we outline our argument, we would like to remark that terminology has connotations that may influence how different types of scientific evidence are valued. Specifically, we find the term ‘actual biological data’ to be problematic because primary data in the biological research domain is generated either from wet-lab experiments or computational simulations, both of which have their idiosyncrasies relative to the underlying biology (Leek *et al.*, 2010). The aim, and often the very purpose, of computational methodology in bioinformatics is to model biological phenomena at a resolution and scale that transcends the current experimental state-of-the-art, to prepare for the arrival of biological data generation at a scale that allows better coverage of biological phenomena. Thus, a term such as ‘experimental calibration’ may be more appropriate to describe the use and purpose of experimental data in the majority of the bioinformatics literature, as opposed to the currently predominant denomination of ‘experimental validation’ (Jafari *et al.*, 2021).

Here, we argue that in the majority of bioinformatics settings, available experimental data do not have the size, resolution and sufficient set of controls (hereafter referred to as ‘limited data’) that would allow for rigorous method assessment. With limited data, performance estimates may be uncertain and sensitive to external factors such as parameter choices. This makes it challenging to judge whether observed improvements over previous methods are substantial, that is, biologically relevant, or merely the result of deliberate tuning of a method to perform particularly well on the experimental dataset(s) at hand (Castaldi *et al.*, 2011; Salzberg, 1997). As a reviewer or critical reader, it is usually unfeasible to generate corroborating (or falsifying) experimental data with similar properties and thus not possible to rule out chance or tuning. Therefore, we argue that increased emphasis on experimental data may lead to insufficient and potentially misleading method evaluation.

In contrast, simulation enables the generation of datasets of virtually unconstrained size, with precise control over introduced signals (ground truth) (Morris *et al.*, 2019). This confers a critical reader the competence to challenge a reported assessment (Meyer and Birney, 2018) and rule out chance results and inappropriate tuning by simply generating new data from the same simulation process, thereby ensuring that conclusions can be meaningfully reproduced and cover a biologically relevant parameter range. Additionally, the specification of a simulation algorithm makes data assumptions for a method explicit and thus contributes to the transparency of a method both in terms of advances over the state of the art as well as its limitations. For example, in immunoinformatics of adaptive immunity, natural immune receptor sequence diversity, which is of the order of >10^13^, is routinely modeled using simulation frameworks for testing biological assumptions and the benchmarking of novel methods (Davidsen *et al.*, 2019; Marcou *et al.*, 2018; Pavlovic *et al.*, 2021; Safonova *et al.*, 2015; Weber *et al.*, 2020).

We stress that simulated data are only meaningful for bioinformatics method development and assessment if it reflects method-relevant underlying biology. The same criterion should be applied to experimental data. We agree that, if available at a sufficient scale, resolution and quality, experimental data are unsurpassed for assessing the capacity of bioinformatics methods to handle the types of signal complexities and data distributions that distinguishes bioinformatics method development from general informatics. Thus, novel methods should indeed be required to be evaluated on experimental data in domains where the available data are sufficiently robust to admit a rigorous assessment. However, we have several concerns with the quality of typically available experimental data for assessment purposes. A first concern is one of size. The performance of a method on a given dataset is always an uncertain estimate of its true performance on the underlying distribution that the observed data reflects. For many biological problems, available experimental datasets are so small that estimate uncertainties can easily be larger than any performance differences observed between competing methods. We fear that a strict journal requirement of employing experimental data may push authors to draw unwarranted conclusions from too small datasets and that reviewers may allow this to pass through due to the lack of good alternatives for the authors. An author’s requirement to include at least rudimentary measures of uncertainty for any reported performance measurement could alleviate these concerns (Walsh *et al.*, 2021). A second concern is that experimental data are often available only for one particular problem setup. This leaves no opportunities to test the sensitivity of a method to variation in problem configuration (to assess how broadly it generalizes) or to test how it performs on data outside the training distribution (whether it is robust to domain shift). A third concern is that there is no possibility to know whether the patterns that a method extracts from experimental data reflect underlying causal relations or not. Furthermore, suboptimal study designs may introduce spurious correlations in datasets, and there is a risk that the best-performing methods at least partly exploit such artificial data patterns.

Also, we fear that a strict, general requirement to assess novel methodology on experimental data may impede progress in method development in the many domains where available data are scarce. In data-scarce domains, we hold that authors should instead be urged to provide a rigorous assessment on simulated data, where authors should explicitly argue for the biological relevance based on either underlying mechanistic knowledge or by calibrating their simulation procedure with experimental data. In particular, we consider such experimentally calibrated simulation to often provide a better assessment of the capabilities of a method than the (in our opinion) too common reliance on anecdotal findings on small experimental datasets, which may reveal more about the ingenuity of the authors than of the proposed method. The discovery of novel biological knowledge does not in itself establish the usefulness or novelty of a new bioinformatics method—it is merely a corollary of, for example, a new method’s greater sensitivity, applicability to a wider parameter range or scalability. While it may be tempting to boost impact by combining novel methodology and novel biological findings in the same paper, this interferes with the assessment of methods on their own merit and thus undermines the selection pressures for the evolutionary process of method improvement in the field.

Our view on the complementarity of simulated and experimental data is in line with the approach to method assessment taken in the machine learning field. Here, the evaluation of simulated data has always had a prominent role. Nowadays, the availability of large and well-curated databases such as ImageNet (Deng *et al.*, 2009) and MNIST (Deng, 2012) make it natural to expect that novel methodologies are also assessed in such real-world data collections. However, when for instance the long short-term memory model was introduced in 1997 (Hochreiter and Schmidhuber, 1997), the authors explicitly asked in their paper ‘which tasks are appropriate to demonstrate the quality of a novel long-time-lag algorithm’ and answered their question based on a collection of exclusively synthetic datasets. Years later, improved data availability revealed that the model is indeed able to learn relevant patterns in a wide variety of real-world domains. The top-cited paper of the present journal (according to ISI web of science) (Li and Durbin, 2009) contains two sections in the Results section entitled ‘Evaluation on simulated data’ and ‘Evaluation on real data’.

While we suggest that well-argued exceptions to the inclusion of experimental data assessment should be allowed for bioinformatics methods research, we can hardly think of any circumstance with compelling reasons for not including any assessment on simulated data. Since method developers should always have a conscious relationship to the data assumptions that they build their models and algorithms on, it should usually be straightforward to implement a simulation of data according to these same assumptions. This allows method developers to confirm that their method behaves as expected (e.g. identification of any bugs), it reveals to developers and readers the range of data parameters within which the method provides sensible results (method transparency) and allows developers or readers to reproduce assessments under identical or modified data assumptions (method reproducibility). We thus encourage basic assessment on simulated data to be considered an integral part of good bioinformatics method craftsmanship. Once simulated data have shown that a method works as intended, experimental data may be used to show that the software works on the field-specific experimental data formats and, ideally, recovers orthogonally validated biological or technological signals.

## 2 Conclusion

We have argued that simulated and experimental data should be considered complementary and of equal importance for assessing methods in typical bioinformatics settings. They should both be strongly encouraged as part of a rigorous review process of novel methodology, where reviewers should ensure that a given paper exploits the best available data sources for assessment (be it experimental or well-established simulated datasets) and when necessary combines data sources for a comprehensive assessment. When available in high quantity, fidelity and generality, experimental data may ensure assessment validity—that a method handles relevant signals and noise profiles from the biological domain. But for many bioinformatics application areas, experimental data are not available at sufficient scale or annotation quality to allow conclusive assessment. Through full control over ground truth and unconstrained data size, simulated data may ensure assessment reliability—that the reported performance of a given method is representative and can be reproduced under the same or modified assumptions of the underlying data generating process. Importantly, sophisticated simulation processes, where signals and noise are calibrated by experimental data or knowledge of underlying mechanisms (Cao *et al.*, 2021; Prakash *et al.*, 2021; Schuler *et al.*, 2017), allows methodology to be developed, assessed and improved early in a field so as to reach a good level of maturity at the time large-scale experimental data starts to become available. Well-calibrated simulation schemes may even be used to explore targeted hypotheses relating to complex biological systems in a way that can guide future experimental data collection (Azencott *et al.*, 2017). In addition, simulation makes explicit the assumptions and layers of biological complexity understood so far and helps identify methodological errors or software bugs.

In summary, we suggest that new bioinformatics methods should be shown to perform comparatively well on ground truth data of a size that allows reliable assessment, be it experimental or simulated. Method developers should be encouraged to make use of both simulated and experimental data, in complementary ways, to cover the multiple purposes of method assessment. When certain roles of assessments are not fully covered, method developers should be expected to provide compelling, explicit reasons—be it reasons for not including assessments involving simulated or experimental data.

## Data Availability

No new data were generated or analyzed in support of this research.
